# Malware Detection of Hangul Word Processor Files Using Spatial Pyramid Average Pooling

**DOI:** 10.3390/s20185265

**Published:** 2020-09-15

**Authors:** Young-Seob Jeong, Jiyoung Woo, SangMin Lee, Ah Reum Kang

**Affiliations:** 1 Department of Future Convergence Technology, Soonchunhyang University, Asan 31538, Korea; bytecell@sch.ac.kr (Y.-S.J.); jywoo@sch.ac.kr (J.W.); 2 Electronics and Telecommunications Research Institute, 218, Gajeong-ro, Yuseong-gu, Daejeon 34129, Korea; sangm@etri.re.kr

**Keywords:** malware detection, Hangul Word Processor, HWP, spatial pyramid pooling, spatial pyramid average pooling, convolutional neural network, stretch padding

## Abstract

Malware detection of non-executables has recently been drawing much attention because ordinary users are vulnerable to such malware. Hangul Word Processor (HWP) is software for editing non-executable text files and is widely used in South Korea. New malware for HWP files continues to appear because of the circumstances between South Korea and North Korea. There have been various studies to solve this problem, but most of them are limited because they require a large amount of effort to define features based on expert knowledge. In this study, we designed a convolutional neural network to detect malware within HWP files. Our proposed model takes a raw byte stream as input and predicts whether it contains malicious actions or not. To incorporate highly variable lengths of HWP byte streams, we propose a new padding method and a spatial pyramid average pooling layer. We experimentally demonstrate that our model is not only effective, but also efficient.

## 1. Introduction

Malware describes malicious software designed for attacking machines in various ways. It may slow down or shut down machines, and often steals or encrypts important files for ransom. Malware can be divided into two categories: malware of executables (e.g., EXE files) and malware of non-executables (e.g., Portable Document Format (PDF) files). Ordinary users are more vulnerable to non-executables because they simply open infected documents without much worry. Although many options have been proposed for the detection of the malware within non-executables, it is still necessary to develop more advanced detection models because new malware for non-executables keeps appearing.

Hangul Word Processor (HWP) is text editing software provided by Hancom Inc., South Korea. HWP is one of the most widely used pieces of software in South Korea and is mainly used in schools, companies, military agencies, and governmental institutions. Due to the relationship between South Korea and North Korea, most malware attacks for HWP files are created by North Korea [1,2]. The HWP files belong to non-executables, so many people in schools and governmental institutions are exposed to threats of malicious HWP files. The malicious HWP files contain byte streams of executable code, shell code, or script code. The byte streams with malicious actions convey different patterns compared to benign byte streams, so it is possible to detect malware by analyzing the byte stream patterns as described in [3].

Meanwhile, many studies have tried to detect malicious actions by applying machine learning models to the byte streams. Such methods usually involve a set of carefully designed features which are then passed to various machine learning models (logistic regression or a support vector machine (SVM) [4]). These studies have a common limitation in that they require substantial effort from experts to define features for different target files (e.g., PDF documents, HWP documents, Word documents); moreover, they require substantial effort regarding the feature definitions whenever new malware appears. Deep learning models have recently been drawing attention because of their ability to automatically extract features from data and because they usually exhibit better performance (e.g., accuracy) compared to traditional machine learning models. Some studies used deep learning models to extract meaningful features from byte streams to accurately detects malicious actions. These studies have a common limitation in that their models are not efficient [3,5,6,7]. That is, the models are often too complex, so they take a long time to analyze numerous suspicious files. As we encounter many suspicious files every day, more efficient (i.e., less complex) models are preferable.

In this paper, we design a convolutional neural network (CNN) to detect malicious actions within HWP files. The CNN model has two newly proposed parts: (1) we propose a new method of padding, namely, stretch padding, and (2) spatial pyramid average pooling (SPAP), which is a variant of spatial pyramid pooling (SPP) [8]. We show that our CNN is efficient and effective through experimental comparisons with other state-of-the-art models.

The rest of this paper is organized as follows. Section 2 reviews previous studies related to malware detection from various different perspectives. Section 3 provides details about the proposed model and compares its structure with other models. Section 4 shows the experimental results and comparisons with other state-of-the-art models. Section 5 provides discussion about the efficiency and overall effectiveness in terms of false positives and false negatives. Finally, Section 6 summarizes and concludes the paper.

## 2. Related Work

### 2.1. Static Analysis of Non-Executable Malware

As the interchange rate of data continues to grow, so does the threat of malware. Malware exhibits malicious behaviors (e.g., destroying files or encrypting files for ransom) and may cause serious damage to individuals, institutions, or companies by stealing or disturbing important information (e.g., contract documents). Malware can be divided into two types: executables and non-executables. Many existing security services (e.g., Norton [9] and Kaspersky [10]) are capable of detecting malware within executables, but the malware in non-executables (e.g., HWP documents) often bypasses security services because such non-executables are ever-changing. Although security services are making attempts to address this, they mostly suffer from a high number of false positives. Many ordinary users tend to open non-executables without much consideration, making them more dangerous.

The non-executables are not executable by themselves, but can be opened or run using the corresponding software (e.g., HWP software, PDF viewer). We have two ways of analyzing non-executables: dynamic analysis and static analysis. Dynamic analysis is used to determine the existence of malicious actions by looking at all of the step-by-step actions of the corresponding binary run in an isolated virtual environment (e.g., virtual box). Kolosnjaji et al. [11] utilized application programming interface (API) call sequences as features and employed long short-term memory (LSTM) [12] with convolutional filters for malware type classification. Xiao et al. [13] defined features using the behavioral patterns of the binary run and applied them to stacked auto-encoders (SAE) [14] for malware detection. These dynamic analyses require a virtual environment to simulate executables, and different studies usually employ different non-public emulation environments; this makes it difficult to reproduce the previous works of dynamic analyses. On the other hand, static analysis finds malicious actions by looking for signatures within the files without executing them. This approach does not require executing the suspicious files, so it is easier to reproduce and compare different models.

### 2.2. Byte Stream of Hangul Word Processor Files

New malware associated with Hangul Word Processor (HWP) files keeps appearing and many users are vulnerable to such attacks because the HWP files are non-executables. Many people recognize that executable files such as portable executable (PE) format files are not secure. However, the security consciousness of document files is relatively weak. Document files are often used for targeted attacks of intelligent malware called advanced persistent threat (APT) attacks. Malicious code is inserted into the document file and attached to the email. At the moment the user opens the document file, the malicious code is executed.

HWP files are used more often by the Korean government than Microsoft office files, so hackers often exploit malicious HWP files for cyber attacks with political purposes. In addition, hackers are making various attempts to utilize existing well-known vulnerabilities such as common vulnerabilities and exposures (CVE) in Microsoft Office files, PDF files, Rich Text Format (RTF) files, and HWP files. In many cases, other vulnerabilities for different document types do not work properly in HWP, but the potential for malware execution still exists. CVE-2014-1761, for example, is a vulnerability in RTF, which has been included in the PrvText stream in HWP. As another example, CVE-2017-11882 is a vulnerability in Microsoft Office Word. In some cases, this vulnerability is included in the stream of the BinData storage in HWP, and is obviously executable, so all streams must be inspected to check whether they contain malicious code.

HWP files have a compound file structure and known as Object Linking and Embedding (OLE). OLE is a file format used in various word documents and is composed of storage and streams similar to the File Allocation Table (FAT) file system. These file types are easy to understand, assuming that storage is similar to folders and that streams are similar to files. Table 1 shows the general structure of an HWP file. In one stream, data are stored in a binary or record structure, and then compressed or encrypted depending on the stream. All data are contained in the stream, alongside malicious code contained in the stream. Checking all streams is equivalent to checking all data constituting the HWP file. If there is an error in the length, compression, or encryption information of each stream, the HWP file cannot be opened normally. In the process of inserting malicious code into the stream, there are many cases in which such information has an error and the HWP file cannot be opened. The stream is composed of several consecutive records, and the record stores header information and data together. The header information includes the type of data, the depth of the hierarchical structure, and the data size. The length of each record is fixed or variable depending on the type of data. BinData storage stores binary data attached to documents, such as images and OLE objects. Streams of BinData or section storage are not limited in size, so there are cases wherein the length is very long.

### 2.3. Neural Networks for Malware Detection

Various machine learning models have been adopted to detect malware in a static manner [15,16]. In Ranveer and Hiray’s work [17], a frequency histogram of pre-defined opcodes was obtained from executables, and was used as a feature vector for malware detection. They used a support vector machine (SVM) and achieved a 0.95 true positive rate (TPR) for the data of VXheavens [18]. Morales-Molina et al. [19] employed a random forest (RF) [20] for malware classification; promising features were selected using principal component analysis (PCA). Darus et al. [21] proposed an approach for malware classification by analyzing grayscale images extracted from Android Package Kit (APK) files. They utilized a GIST descriptor [22] to generate features, and achieved about 70% accuracy. All of these studies have a common limitation in that they require intensive effort for feature engineering; the feature definitions have a huge impact on the final results, thereby requiring a large amount of time to define features carefully for every type of malware.

Deep learning models have recently been drawing attention because they do not require much effort in regard to feature engineering and show better performance (e.g., accuracy) than other machine learning models by automatically extracting promising features. Saif et al. [23] designed a deep belief network (DBN) for malware detection in both static and dynamic ways. The DBN was originally proposed in [24]; it is typically pre-trained using an unsupervised contrastive divergence (CD) algorithm for every pair of adjacent layers, and is fine-tuned using standard back-propagation. They compared the DBN with other models (e.g., SVM, random forest) experimentally, and the DBN achieved the best accuracy of 99.1%. In [25], a recurrent neural network (RNN) [26] was employed to detect malware in a dynamic manner. They took a short snapshot of the behavioral history of portable executable (PE) samples and achieved an accuracy of 94% using RNN with gated recurrent units (GRU) [27]. Yan et al. [5] utilized both an RNN and a CNN [28] for malware detection of executables. At the pre-processing phase, they generated grayscale images from byte streams and extracted opcode sequences. They delivered the grayscale images and the opcode sequences to the CNN and RNN, respectively. The results generated by the two deep learning models were then delivered to a stacking ensemble model that achieved 99.88% accuracy. These studies have a common drawback in that they require hand-crafted features, although deep learning models are known to automatically extract features; the deep learning models may give better results when given carefully designed inputs based on domain knowledge, but these studies have a strong downside, as they require large amounts of effort from domain experts whenever new malware appears. It is necessary, therefore, to develop deep learning models that work with more hands-off approaches toward feature engineering, and using only byte streams may be the best choice in this sense.

There have been few studies on malware detection that used byte streams for training deep learning models. Jeong et al. [6] designed a CNN model for malware detection of PDF files, wherein the input length is assumed to be 1000 bytes. They extracted byte streams from the PDF files and directly fed them to the CNN model. Their CNN model achieved an F1 score of 98.48–98.65%, which was superior to other machine learning models. In [3], a CNN model was proposed for malware detection of HWP files, wherein the input length is assumed to be 600 bytes. This was the first study of malware detection for HWP files using only byte streams, and achieved an F1 score of 93.33–93.45%. Raff et al. [7] designed a shallow structure of a CNN for analyzing byte streams of PE headers. They assumed an input length of 1–2M bytes, in order to resolve the issue of variable length of byte streams. Their proposed model achieved 94% accuracy but suffered from poor efficiency; they had to consider small batch sizes because of the huge amount of trainable parameters of the model. These studies commonly employed a CNN rather than other deep learning models (e.g., RNN or DBN) because CNN has relatively smaller number of parameters and is known to be effective for capturing local patterns.

The previous studies that applied a CNN model to byte streams were successful in some sense, but may not be useful when the byte streams are very long. For example, we may have to run the model proposed in [3] numerous times (about hundreds times) to make a decision for a single file. Indeed, we found that the mean stream length of PDF files of [6] is about 600, whereas the mean stream length of HWP files used in [3] is about 350,000–710,000 with a standard deviation of 2,000,000–4,000,000. This implies that we may need to run the model of [3] about 580–1180 times for each byte stream, as it is assumed that the input length is 600 bytes. Moreover, if a target HWP file has multiple byte streams, then we may need to run the model tens of thousands of times. The model proposed in [7] has a shallow convolutional structure to cover 1M–2M byte streams. However, the core of this structure is just a couple of standard convolutional layers together with a global max-pooling layer, and thus it has no way to incorporate the highly variable stream lengths of HWP files; the length of HWP byte streams has a high standard deviation, so the model of [7] is generally poor at capturing important local patterns and suffers from low performance in terms of effectiveness (e.g., F1 score).

In this paper, we design a CNN for malware detection that works in a static manner. The model takes byte streams as input and predicts whether the corresponding byte stream has malicious actions or not. This model has an extremely small number of parameters, but we will show that the model achieves high efficiency (e.g., number of parameters) and effectiveness (e.g., F1 score). We believe that our proposed model is practically useful because it works fast without requiring highly specific machines and gives accurate results.

## 3. Proposed Method

The purpose of this study to develop a CNN model for malware detection of HWP files. We designed the CNN model with two requirements in mind: (1) it had to take long byte streams as input so that we do not have to run the model numerous times for decision making, and (2) it should be as light as possible. It is difficult to satisfy both requirements; if we lean too much to the first requirement, then we may completely lose the second one. The first requirement allows the model to be easily applicable to any newly appearing piece of malware, while the second requirement is associated with the efficiency (e.g., the number of parameters) of the model. The model, of course, should be effective (e.g., high accuracy) as well, but will be ultimately useless if it works too slowly. Therefore, the model should be both efficient and effective.

**Figure 1:**
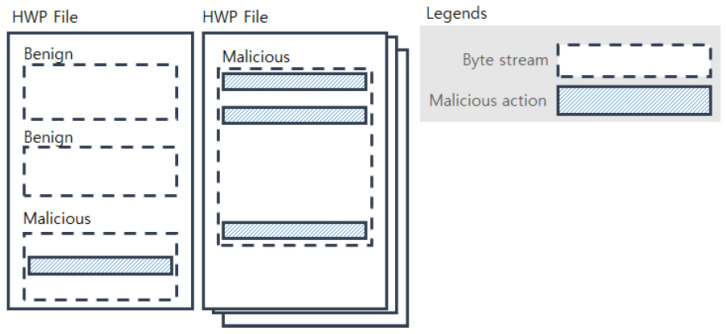
Malicious/benign byte streams within HWP files.

**Figure 2:**
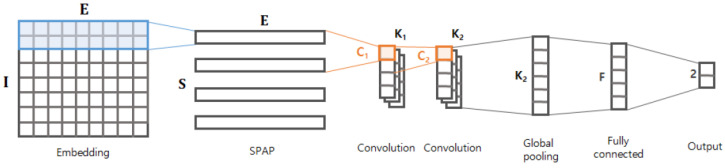
Graphical structure of the proposed model.

Figure 1 shows examples of malicious and benign byte streams within HWP files; if a byte stream has at least one malicious action, then it is considered a malicious byte stream. Different HWP files typically have different numbers of byte streams, and the byte streams have variable lengths. Although malicious actions are known to be 600 bytes or shorter in length, as reported in [3], a model taking a longer byte stream will be preferable because the byte streams of HWP files have much longer lengths (e.g., 350,000–700,000) than PDF files. Our model is designed to deal with such long streams and its graphical representation is depicted in Figure 2.

The first layer is the embedding layer, which projects each byte of the stream into a vector of a certain dimension *E*. As described in [7], a byte is just a categorical value, so it is not proper to take the raw byte as an input. The embedding layer converts every byte into a vector with meaningful representation, which in turn helps the following layers better comprehend or extract arbitrary patterns from the given byte stream. In this study, the embedding layer was trained on our data.

If an input is a byte stream of length *I*, then it will be transformed into an I×E matrix via the embedding layer. As the *E*-dimensional transformed vector conveys semantic patterns of the corresponding byte, semantically related (or similar) bytes would have small distance between them.

**Figure 3:**
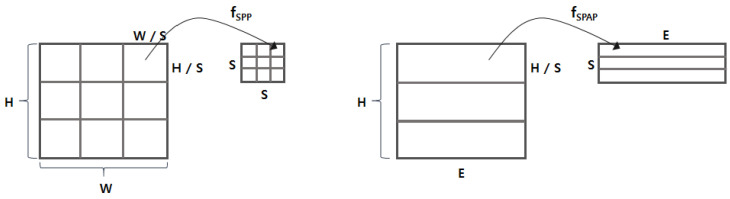
Comparison between the SPP and the SPAP. (**left**) The SPP working on a two-dimensional image; (**right**) the SPAP working on a stream of embedding vectors.

The output matrix of the embedding layer is delivered to the spatial pyramid average pooling (SPAP) layer. We next designed the SPAP layer, which is a variant of a spatial pyramid pooling (SPP) layer [8]. The SPP layer allows for generating an output of the same size by picking an item from S×S divided regions even with different input sizes. There are two key differences between SPAP and SPP: (1) the SPP runs in two dimensions, whereas the SPAP runs in a single dimension; and (2) the SPP basically considers a max-pooling, whereas the SPAP takes an average-pooling. The SPP layer was originally designed for image processing, so it assumed that the input is a two-dimensional matrix (e.g., height *H* and width *W*). As shown in Figure 3, the SPP layer generates the output by applying the function $f_{SPP}$ to every region of W/S×H/S elements (e.g., pixels). In contrast, the SPAP layer takes a stream of *I* embedding vectors and generates the output by applying the function $f_{SPAP}$ to every I/S adjacent embedding vector. Furthermore, the $f_{SPP}$ for the SPP layer is basically a max-pooling function that picks the biggest value (i.e., the brightest pixel), but the $f_{SPAP}$ is an average-pooling function, as defined in Equation (1), where $e_{i}$ represents the *i*-th embedding vector. Adopting the average-pooling function is crucial because the input consists of not just values (e.g., pixel intensity) of the region, but also includes the embedding vector conveying semantic information. As described in [29], the averaging embedding vectors may find deeper semantic information (i.e., relational information) between the given adjacent embedding vectors; we demonstrated its validity experimentally.

(1) $$f_{SPAP}\left(e_{1},e_{2},\ldots ,e_{W/S}\right):=\frac{1}{W/S}\sum_{i}e_{i}$$

The S×E output matrix is passed on to two consecutive convolutional layers as described in Figure 2. The first convolutional layer takes the $C_{1}$ adjacent embedding vectors and generates outputs via $K_{1}$ channels; similarly, the second convolutional layer generates outputs via $K_{2}$ channels based on the $C_{2}$ adjacent values of the previous layer. Adopting the two consecutive layers is inspired from [3], where the consecutive layers were found to effectively capture local semantic patterns of the byte sequence. The output of the second convolutional layer is delivered to the global max-pooling (GP) layer. Using the GP layer is inspired from [7]; it is known to dramatically reduce the number of parameters without losing important information. The GP layer is followed by a fully-connected (FC) layer of *F* nodes, and finally it ends with the output layer. The output layer has two nodes: a benign node and a malware node. Thus this model solves a binary classification.

**Figure 4:**
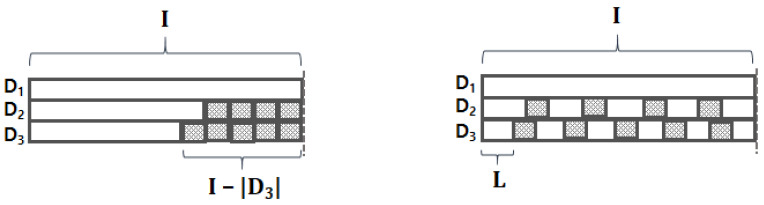
Comparison between two padding methods, where the shaded squares represent padding tokens. (**left**) Conventional padding. (**right**) Stretch padding.

When we train the model, we first need to ensure the input data have equal shape; in other words, every byte stream has to be equal in length. The most widely-used method for this is a padding. As shown in the left figure of Figure 4, conventional padding is used to attach some special-purposed tokens (e.g., padding token) to the tail (or front) of a sequence. For example, if we need to make all byte streams have the same length of *I*, then the third byte stream $D_{3}$ will be padded with $I-|D_{3}|$ special tokens at its tail. However, this may result in important information loss since our input length has high variance (e.g., σ = 3,977,756 for benign HWP streams); such high variance may cause pooling operations on embedding vectors of only padding tokens. To alleviate this “pooling on padding tokens” problem, we propose “stretch padding,” which stretches the existing elements of the vector to have the desired length *I*. As shown in the right figure of Figure 4, the byte streams are stretched to have equal length by interleaving padding tokens within the stream; this helps prevent the “pooling on padding tokens” problem. We demonstrate the impact of the stretch padding experimentally in the following section.

## 4. Experimental Results

We compared our model with other state-of-the-art models experimentally. We considered the HWP dataset used in [3], which is the largest public dataset of HWP files as far as we know. The dataset contains 534 HWP files (benign: 79; malicious: 455); the imbalance between the two classes (e.g., benign and malicious) is for incorporating diverse attack strategies among the malicious HWP files. As described in Figure 1, there might be one or more malicious byte streams within a malicious HWP file. Thus, we generated benign and malicious stream samples using Algorithm 1 of [3] with the same parameters, but with the input length of 100,000. The training dataset and the test dataset were sampled from different files. The stream samples were divided into a training set and a test set, and their statistics are summarized in Table 2. With the stream samples, all experiments were conducted using a machine with an Intel(R) Core(TM) i7-9800 CPU 3.80 GHz, four Geforce RTX 2080 Ti, and 64 GB RAM. We implemented the model using Tensorflow Keras 1.13.

Table 3 shows the experimental results for efficiency, where Cons-Conv, Mal-Conv, and SPAP-Conv indicate [3,7], and our model, respectively. The Cons-Conv was originally designed with the assumption that the input length is 600, and the Mal-Conv assumed that the input length is 1M–2M. For fair comparison, we set them to have the same input length of *I* = 100,000. Except for the input length *I*, the Cons-Conv and Mal-Conv followed their original structures. For the SPAP-Conv, we set *S* = 512, *E* = 8, $K_{1}$ = 64, $K_{2}$ = 256, $C_{1}$ = 3, $C_{2}$ = 3, and *F* = 64. We applied batch normalization [30] with a momentum of 0.99 and a leaky Relu activation function [31] with α = 0.3 to the two convolutional layers. The fully-connected layer is followed by a Relu activation function and dropout with a probability of 0.5. The output layer is followed by the softmax function. We used a cross entropy function as the loss and Adam’s optimizer [32] with an initial learning rate of 0.001. Similarly to SPAP-Conv, the batch normalization and dropout were applied to Cons-Conv, whereas Mal-Conv was trained without batch normalization since batch normalization is known to be not helpful to Mal-Conv, as reported in [7]. SPAP-Conv was trained for five epochs, and the two other models were trained for 10 epochs.

The three models are compared according to the number of trainable parameters (#Params), floating-point operations per second (FLOPS), and runtime for the test set in seconds (Runtime). It is obvious that smaller #Params, FLOPS, and runtime are better; smaller #Params and FLOPS will allow the model to work without highly specific machines, while a smaller runtime will reduce the time needed for the malware detection process. Cons-Conv turned out to be the heaviest and slowest model; the runtime for 652 byte streams was 15.8662 seconds, meaning it may take several minutes if we need to analyze multiple HWP files with 20–30 byte streams. Mal-Conv is about half the size compared to Cons-Conv in terms of #Params and FLOPS, but is much faster than Cons-Conv. The reason for this might be that the heavy structure of Cons-Conv caused a hardware-level delay (e.g., virtual memory or fragment memory) as reported in [33], so the runtime gap is much greater than the size gap. Meanwhile, SPAP-Conv is the best in terms of size; SPAP-Conv is about 15 times lighter than Mal-Conv. However, We observed that Mal-Conv has a comparable runtime to SPAP-Conv. This is because Mal-Conv was implemented using public Keras layers (e.g., Conv2D layer and dense layer), whereas SPAP-Conv had our implemented SPAP layer which is not provided by Keras. We used the “slicing” operation to implement the SPAP layer; the tensorflow version of this operation is known to be about 300 times slower than other alternative (e.g., numpy); in short, this is an implementation issue. That said, it is obvious that SPAP-Conv works quickly as it needs only 1.5 milliseconds to analyze a byte stream.

Table 4 summarizes the results regarding effectiveness. This table has two parts: the results of our proposed stretch padding and the results of conventional tail padding. The three models improve with stretch padding; these models are commonly CNN models, and thus benefit from stretch padding because such padding helps to prevent the “pooling on padding tokens” problem by making the sizes of the padding chunks small. If the byte stream is extremely short (e.g., 1–10 bytes), then the sizes of the padding chunks may be too big (e.g., 10,000 bytes). In such cases, it will be better to consider overlapped byte streams. For example, if we take overlapped byte streams of length 3 for a given byte stream [A,B,C,D], then we will have [A,B,C] and [B,C,D].

Among the three models, Cons-Conv achieved the best recall of 94.79 for the malicious case. As our focus is the malware detection problem, Cons-Conv might be the best option if we wish to never miss malicious streams. However, its precision for the malicious case and its F1 score were the worst of the three models; moreover, it was the worst model in terms of efficiency, so it will not be good for practical usage. On the other hand, SPAP-Conv achieved the best F1 score with stretch padding and its recall for the malicious case was not much poorer compared to Cons-Conv. Furthermore, it is the lightest model among the three and its runtime is much shorter than that of Cons-Conv.

## 5. Discussion

Based on the results concerning effectiveness, SPAP-Conv is the best among the three models. Note that SPAP-Conv is about 15 times lighter than Mal-Conv, but SPAP-Conv achieved a greater F1 score than Mal-Conv. As the runtime of SPAP-Conv is also fast, we believe that it will be the best for practical usage. It is also worth noting that SPAP-Conv has the largest F1 score gap between stretch padding and tail padding. This implies that the SPAP layer is the appropriate layer for gathering important information given by stretch padding.

One may argue that the average pooling of the SPAP layer might not be very helpful. To respond to this, we replaced the average pooling of the SPAP layer with conventional max pooling and provide the results in Table 5. By comparing these results with Table 4, we observe that max pooling degrades the F1 score compared to SPAP-Conv with tail padding. This implies that the average pooling plays a crucial role in the SPAP layer, which is consistent with [29]; that is, averaging the embedding vectors helps to find deeper semantic information among the embedding vectors.

High recall in the malicious case indicates that malware is detected without omission, and the best model for this is Cons-Conv with a recall of 94.79% for the malicious case. However, Cons-Conv may suffer from false positives because its precision in the malicious case was only 85.65%, which was the worst among the three models. As described in [34], a high risk of false positives affects the security software development process and can lead to loss of business. On the other hand, Mal-Conv was the best in terms of false positives, with a precision of 98.58% for the malicious case. Mal-Conv, however, may suffer from false negatives because its precision in the benign case was only 81.61%. Mal-Conv also has the worst recall in the malicious case of 86.35%. SPAP-Conv had the best F1 scores for both the benign and malicious cases, making it a suitable point of compromise between false positives and false negatives.

## 6. Conclusions

We designed a new CNN model for malware detection by analyzing byte streams within HWP files. To incorporate the highly variable length of HWP byte streams, we propose a new padding method, stretch padding, in conjunction with an SPAP layer. We experimentally demonstrated that stretch padding together with the SPAP layer improves the performance (e.g., F1 score). Compared to other recent models, we showed that the proposed model is not only effective, but also efficient. In future studies, we will consider more malicious HWP files and investigate better model structures; for example, we will check whether a deeper SPAP layer is helpful or look for a more shallow structure to obtain better efficiency.

## Figures and Tables

**Table 1:** Structure of HWPfiles.

Type	Name (*Storage or Stream)	Length
File recognition information	FileHeader	fixed
Document information	DocInfo	fixed
	*BodyText	
Main document	Section 0	unfixed
	Section 1	
Document summary	HWPSummaryInformation	fixed
	*BinData	
Binary data	BinaryData0	unfixed
	BinaryData1	
Preview text	PrvText	fixed
Preview image	PrvImage	unfixed
	*DocOptions	
Document options	LinkDoc	unfixed
	DrmLicense	
	*Scripts	
Script	DefaultJScript	unfixed
	JScriptVersion	
	*XML Template	
XML template	Schema	unfixed
	Instance	
	*DocHistory	
Document history	VersionLog0	unfixed
	VersionLog1	

**Table 2:** Statistics of sampled data.

	Total	Malicious	Benign
Train + Test	6520	3668	2852
Train	5868	3265	2603
Test	652	403	249

**Table 3:** Experimental results about efficiency, where #Params indicates the number of trainable parameters, FLOPS stands for floating-point operations per second, and Runtime is the time spent for running the model on the test set in seconds.

	#Params	FLOPS	Runtime
Cons-Conv	2,056,354	4,112,896	15.8662
Mal-Conv	1,043,074	2,085,384	0.8572
SPAP-Conv	70,274	143,453	0.9831

**Table 4:** Experimental results about effectiveness, where the two values *b*/*m* of each cell correspond to benign and malicious cases, respectively.

	Model	F1 (%)	Precision (%)	Recall (%)
stretch	Cons-Conv	81.32/89.99	89.81/85.65	74.30/94.79
	Mal-Conv	89.05/92.06	81.61/98.58	97.99/86.35
	SPAP-Conv	92.86/95.08	87.28/99.46	99.20/91.07
tail	Cons-Conv	80.00/88.54	84.07/86.15	76.31/91.07
	Mal-Conv	86.83/90.72	80.69/95.86	93.98/86.10
	SPAP-Conv	86.96/92.33	89.74/90.67	84.34/94.04

**Table 5:** Effectiveness of the SPAP-Conv with max pooling, where the two values *b*/*m* of each cell correspond to benign and malicious cases, respectively.

	F1 (%)	Precision (%)	Recall (%)
SPAP-Conv with max-pooling	84.67/92.27	98.40/86.21	74.30/99.26
